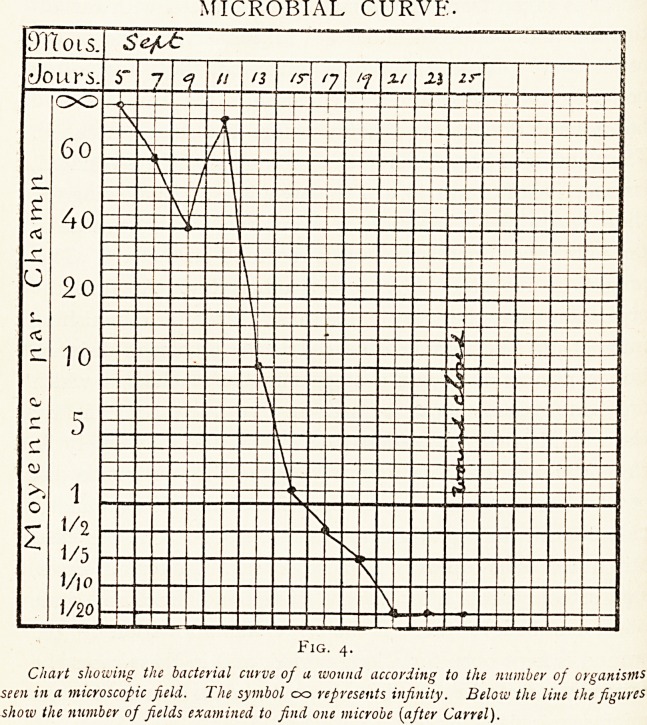# The Prevention of Sepsis in War Wounds, with Special Reference to the Carrel-Dakin Method

**Published:** 1917-07

**Authors:** James Swain

**Affiliations:** Professor of Surgery in the University of Bristol. Lieut.-Colonel, R.A.M.C.: Consulting Surgeon to the Southern Command; a British Delegate to the Inter-Allies Congress on the Treatment of Wounds in War


### THE PREVENTION OF SEPSIS IN WAR WOUNDS, WITH SPECIAL REFERENCE TO THE CARREL-DAKIN METHOD.

BY

JAMES SWAIN, C.B., M.S., M.D. Lond., F.R.C.S.,

*Professor of Surgery in the University of Bristol.*

*Lieut.-Colonel, R.A.M.C.: Consulting Surgeon to the Southern Command; a British Delegate to the Inter-Allies Congress on the Treatment of Wounds in War.*

DURING my visits to France I have seen many hospitals in the English and French lines and in Paris where the scientific methods with which war wounds are now treated, even within a few miles of the firing-line, are in noticeable contrast with the unsatisfactory procedures adopted at the beginning of the war.

The control of infection and suppuration in war wounds is now almost a matter of mathematical certainty. Microscopic tests as to the progress of the sterilisation of wounds are applied, and the rate of progress of the healing of aseptic wounds can be definitely calculated and represented by a geometrical curve.

At a time when the base hospitals in England were filled with a large proportion of suppurating cases (much of which was due to the re-infection of wounds during transport), it was strange to see printed in large type in M. Tuffier's wards at the Beaujeu Hospital a notice, in French, to the following effect: "Every patient whose wound suppurates has the right to ask the reason of his surgeon." The statement was amply justified, for in this hospital, and elsewhere, pus was practically non-existent, wounds were supple and free from inflammation and pain, compound fractures were healing like simple ones, and empyemata were converted into cases of sterile pneumothorax. Early treatment is highly desirable, and as this can be commenced in France soon after the infliction of the wound, most of the cases are rendered "sterile," and closed, with about 99 per cent. of successes, in the course of five to twenty-five days.

The hospitals to which these remarks refer have adopted the Carrel-Dakin method of treatment, and it is easy to see from what has been said how much suffering is prevented, and how many limbs and lives have been saved by this means of prevention of sepsis.

In order to be successfully applied, great attention to detail is necessary, and as there is considerable misunderstanding on some points, it is desirable to state some of the methods adopted by the staff at Carrel's Hospital at Compiègne, where I was sent by the War Office to study this form of treatment.

The fluid used to "sterilize" the wounds is Dr.

Daufresne's modification of Dakin's solution of hypochlorite of soda. It must be free from caustic soda, and should contain exactly between 0.45 and 0.50 per cent. of hypochlorite of soda—under 0.45 per cent. the solution is not active enough, and over 0.50 per cent. it is irritant.

The solution is made by mixing chlorinated lime (bleaching powder), dry carbonate of soda and bicarbonate of soda in water; but as the amount of active chlorine in bleaching powder varies within wide limits, it is necessary to titrate the bleaching powder before use.

The following instructions for titrating the chlorinated lime and preparing the solution are taken from a paper supplied from the laboratories of the Rockfeller Institute at Compiègne :—

*Technique of the titration of chlorinated lime* (*bleaching powder*).—Obtain an average sample of the bleaching powder by mixing small quantities obtained from different parts of the material. Weigh 20 grammes of this, mix it thoroughly in a litre of water, and allow it to stand for some hours. Filter.

Measure exactly 10 c.c. of the clear fluid with a graduated pipette, and add 20 c.c. of a 10 per cent. solution of iodide of potassium, and 2 c.c. of acetic acid or hydrochloric acid. To this mixture add, drop by drop, a decinormal solution of hyposulphite of soda until decoloration is complete. The number of cubic centimetres of the solution of hyposulphite of soda required for decoloration, multiplied by 1.775, will give the weight of active chlorine contained in 100 grammes of bleaching powder.

This figure being known, the following table can then be used to give the quantities of chlorinated lime (bleaching powder), carbonate of soda, and bicarbonate of soda, which should be used to prepare 10 litres of Daufresne's solution :—

| Figure for Active Chlorine per cent. | Quantities to be used to Prepare 10 Litres of Daufresne's Solution at 0.475 per cent. of NaCl.O. | | |
|---|---|---|---|
| | Bleaching Powder. | Dried Carbonate of Soda. | Bicarbonate of Soda. |
| | gr. | gr. | gr. |
| 20 | 230 | 115 | 96 |
| 21 | 220 | 110 | 92 |
| 22 | 210 | 105 | 88 |
| 23 | 200 | 100 | 84 |
| 24 | 192 | 96 | 80 |
| 25 | 184 | 92 | 76 |
| 26 | 177 | 89 | 72 |
| 27 | 170 | 85 | 70 |
| 28 | 164 | 82 | 68 |
| 29 | 159 | 80 | 66 |
| 30 | 154 | 77 | 64 |
| 31 | 148 | 74 | 62 |
| 32 | 144 | 72 | 60 |
| 33 | 140 | 70 | 59 |
| 34 | 135 | 68 | 57 |
| 35 | 132 | 66 | 55 |
| 36 | 128 | 64 | 53 |
| 37 | 124 | 62 | 52 |

Thus, for example, assuming it was necessary to use 16.6 c.c. of the decinormal solution of hyposulphite of soda to obtain decoloration, the percentage of active chlorine in the bleaching powder is 16.6 by 1.775=29.7 per cent. The quantities of the various ingredients for the preparation of 10 litres of the solution, in this case, would be—

Bleaching powder .. .. 154 grammes.
Dry carbonate of soda .. .. 77 grammes.
Bicarbonate of soda .. .. 64 grammes.

And if one has only the carbonate of soda in crystal form, it would be necessary to replace the 77 grammes of dry carbonate by 220 grammes of the crystallised carbonate of soda.

*Preparation of Daufresne's solution* (10 litres).—Weigh exactly the quantities of the various ingredients determined in the manner already described. Put into a 12-litre flask the bleaching powder and 5 litres of ordinary (not distilled) cold water, shake vigorously for a few minutes, and leave for six to twelve hours, one night for example. At the same time dissolve the carbonate and bicarbonate of soda in 5 litres of ordinary cold water. After the lapse of six to twelve hours, pour the salt solution into the flask containing the macerated bleaching powder, shake vigorously for a few seconds and wait to allow the calcium carbonate to be precipitated. After about half an hour siphon the liquid and filter with a double paper to obtain a good clear solution. The solution must be kept in a dark place, as light rapidly destroys the sodium hypochlorite. It is best kept in large, wicker-covered, dark green bottles, and if five milligrammes of potassium permanganate are added to each litre of the solution, the stability of the hypochlorite is greatly enhanced The solution should be neutral, and should give no coloration on being tested by the addition of a few centigrammes of phenolphthalein in powder to 20 cubic centimetres of the liquid.

*Titration of the Daufresne's solution.*—It is desirable to verify the percentage of the solution from time to time. This is done in the same manner as the titration of the bleaching powder: measure ten cubic centimetres of the solution, add twenty cubic centimetres of a 10 per cent. solution of iodide of potassium, and two cubic centimetres of acetic acid; then add, drop by drop, a decinormal solution of sodium hyposulphite until decoloration occurs. The number of cubic centimetres used, multiplied by 0.03725, gives the weight of hypochlorite of soda contained in 100 cubic centimetres of the solution. The Daufresne's solution is of proper strength when it is necessary to use for discoloration 12 to 13 cubic centimetres of the hyposulphite solution. $13 \times 0.03725 = 0.485$ per cent. NaCl.O.

*Method of Application.*—It is unnecessary to describe this in detail, but briefly it may be said that, if possible, within a few hours of being wounded the patient is anæsthetised, foreign bodies and portions of clothing are removed from the wound, which is excised, thoroughly cleansed, laid freely open, and dressed according to the Carrel technique. Tubes (in number according to the size of the wound, of 4 mm. inside diameter, closed at the end, but having numerous fine perforations just above for 5 to 20 centimetres) are placed in the wound, supported loosely by gauze wrung out of Daufresne's solution or fixed in place by strapping. The skin around the wound is protected by pieces of gauze measuring about 6×12 c.m., soaked in vaseline and sterilised, and the whole wound is covered by a sterilised dressing passing round the limb, and consisting of a layer of absorbent wool inside (*i.e.* next to the wound) and a layer of non-absorbent wool outside enclosed in gauze. This pad is fastened with safety pins in such a way that the proximal ends of the Carrel's tubes project through the place of meeting of the edges of the wool dressing. (Fig. 1.[1]) To

FIG. 1.

*The disposition of a 4-way tube at the surface of a dressing (after Carrel).*

[1] Figs. 1, 2 and 4 are taken from *Le traitement des plaies infectées*, par A. Carrel and G. Dehelly. Mazzon et cie, 1917. Fig. 3 is taken from *Pratique de l'irrigation des plaies dans la méthode du Docteur Carrel*, par J. Dumos et Ann Carrel. A. Maloine et Fils, 1917. Figs. 3 and 4 have been slightly altered.

the ends of the tubes glass connections of one to four openings (Fig. 2) according to necessity are fastened, and these glass connections are in turn connected by a rubber tubing, through which the Daufresne's solution runs from a reservoir of a litre in capacity, which is placed not more than three feet above the patient's bed. (Fig. 3.) On this main supply tube a clip is placed close to the reservoir, and the clip is released for about one to three seconds every two hours, so as to flood the wound

FIG. 2.

*Different forms of glass distributing connexions (after Carrel).*

according to its size with 20 to 100 cubic centimetres of the fluid. This flooding of the wound every two hours is sometimes arranged for by automatic mechanical or electrical devices. If the patient complains of pain, the reservoir should be lowered.

The wounds are dressed daily, but the tubes are generally changed on alternate days. When redressing takes place, the skin is cleansed with a solution of 20 per cent. oleate of soda on small swabs of wool. The dressing of these wounds is carried out with the usual antiseptic precautions; the

FIG. 3.

Scheme of general arrangement for the distribution of the fluid (after Carrel).

surgeon works in gloves, and all manipulations are carried out by means of two pairs of sterilised forceps, which are changed for each wound.

When the bacteriological examination (*vide infra*) of the wound shows it to be practically free from organisms, it is closed by suture or strapping without drainage, after excision of the skin edge.

With regard to the preliminary excision of the wound which has been referred to, it should be observed that if a wound can be thoroughly and completely excised within six or eight hours of the receipt of injury, it may be possible to close the wound at once, and get healing by first intention with or without antiseptics. When the wound cannot be thoroughly excised, or when more than eight hours have elapsed after the injury, the bruised or dirty tissues should be cut away as far as possible, the wound opened up, and Carrel treatment commenced at once. If the patient does not come for treatment until after twenty-four to forty-eight hours from the time of injury, Carrel thinks the wound should not be excised. In these last cases there should be as little surgical interference as possible, and foreign bodies are better left in until the Carrel technique has been carried out for some days. In short, surgical cleansing during the pre-inflammatory period should be complete, and is fairly free from danger ; but during the early days of the inflammatory period only such surgical intervention as may be absolutely necessary should be undertaken, as there is great danger of increasing the septic trouble. This risk is much less when suppuration is established.

*Bacteriological Examination of the Wound.*—It is quite impossible to tell the condition of a wound as regards infection from naked-eye appearances.

A microscopic examination of the secretions of a wound is necessary in order to ascertain the degree of infection at the beginning of treatment, to follow the progress of the effect of the antiseptic applied for the purpose of disinfection, and to determine the time at which closure of the wound may be undertaken without danger to the patient.

The bacteriological examination now referred to is merely quantitative, and has nothing to do with the cultural methods employed to ascertain the nature of the infection. A smear is taken from the wound, fixed, stained with carbol-thionin, and examined under a $\frac{1}{12}$ in. immersion lens in the usual way. Carbol-thionin is preferred, but any other non-selective stain will do. Irrigation of the wound should be stopped at least two hours before the smear is taken. The number of organisms in the microscopic field is counted, irrespective of the variety of the organisms. By moving the slide, several fields can rapidly be observed, and the mean of the number of organisms seen is recorded on a chart. This process is repeated about every other day. At first the organisms may be very numerous; later, a diminishing number per field may be observed as the process of disinfection continues. At last a stage may be reached in which only one organism can be seen in five microscopic fields; the wound is then considered "sterile," and may safely be closed by secondary suture or strapping, with almost no risk of any latent sepsis. It has been found that if a wound is closed when the organisms are as numerous as one per field stitch abscesses are very liable to occur. In certain cases, *e.g.* osteomyelitis after severe compound fracture, some observers believe there is better callus formation if the wound is kept open for a long period, and in these cases the organisms may be found to be reduced to one in twenty microscopic fields or less.

The record (Fig. 4) of the number of organisms found—the "courbe microbienne"—is now largely used as an indication of the result of treatment, whether the Carrel-Dakin or other technique is employed. So long as the number of organisms steadily diminishes, treatment may be considered satisfactory; but if the number remains stationary or increases, the cause of the arrest of disinfection should be sought, *e.g.* insufficient number of tubes, presence of sloughs, foreign bodies, pieces of clothing, faults in technique, etc.

MICROBIAL CURVE.

FIG. 4.

*Chart showing the bacterial curve of a wound according to the number of organisms seen in a microscopic field. The symbol ∞ represents infinity. Below the line the figures show the number of fields examined to find one microbe (after Carrel).*

*General Remarks.*—While it should be admitted that we owe a great deal of our knowledge of the physiology of wounds to Sir Almroth Wright, the results of the Carrel-Dakin treatment have abundantly proved the superiority of the antiseptic treatment of wounds over the treatment by saline solution, normal or hypertonic. There are many ways of securing disinfection of wounds, but the method of Carrel seems one of the most satisfactory. On the other hand, there are difficulties in the preparation of the fluid in order to ensure the correct strength, and in connection with the necessity of flushing the wound every two hours, owing to the fact that the hypochlorite of soda so rapidly loses its antiseptic properties when in contact with the tissues. A solution of flavine (a dye of the acridine series) is so easily made of the strength of 1 in 1,000, and appears to act so particularly well in contact with serum, that it has been suggested that this solution might replace the Daufresne's solution in the Carrel technique. There are reasons for believing that the results will not be so satisfactory.

In order to obtain a more abiding antiseptic, and so obviate the necessity of frequently flushing the wound with the Daufresne's solution, experiments are being carried out with a view to perfecting a chloramine paste which at present is in use, of a strength of 1 per cent. of chloramine with a stearate of soda basis. The aromatic chloramines were found by Dakin while he was searching for an intermediate substance upon which he thought the antiseptic action of the hypochlorites depended. So far it may be said that chloramine paste, when applied to a wound, will check the further development of organisms in the wound for twenty-four or forty-eight hours, and is therefore useful for cases which must be transported. At Compiègne superficial wounds were often treated with chloramine paste, and the antiseptic power of the paste appeared to be only slightly inferior to that of the hypochlorite solution.

Possibly future improvements in wound treatment will be found to be associated with some form of paste or fluid, which allows of the continuance of its antiseptic properties for a considerable period, and so saves the labour and pain necessitated by frequent dressings. Morison's bismuth and iodoform paste has already achieved highly satisfactory results, and Mencière uses an embalming fluid, for which he claims success in combating the infection of wounds as shown by the bacterial curve, in the same way as that already described for the Carrel-Dakin technique.

It is desirable to point out that Daufresne's solution should not be heated; it should not be used in the general peritoneal cavity, nor applied to the eye, and it should not be used intravenously as it is hæmolytic, and has thus caused death.

## Figures and Tables

**Fig. 1. f1:**
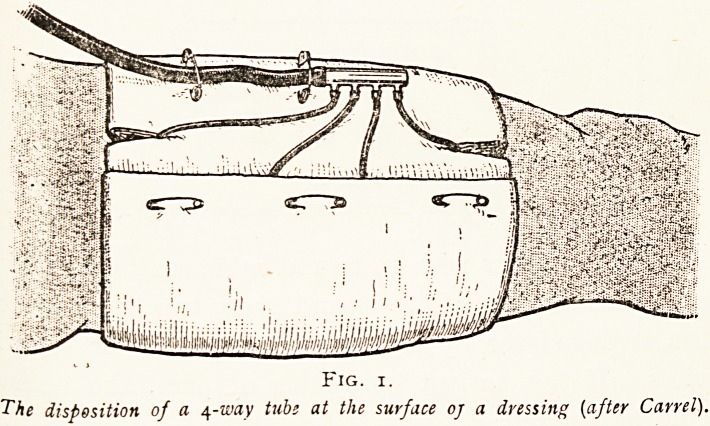


**Fig. 2. f2:**
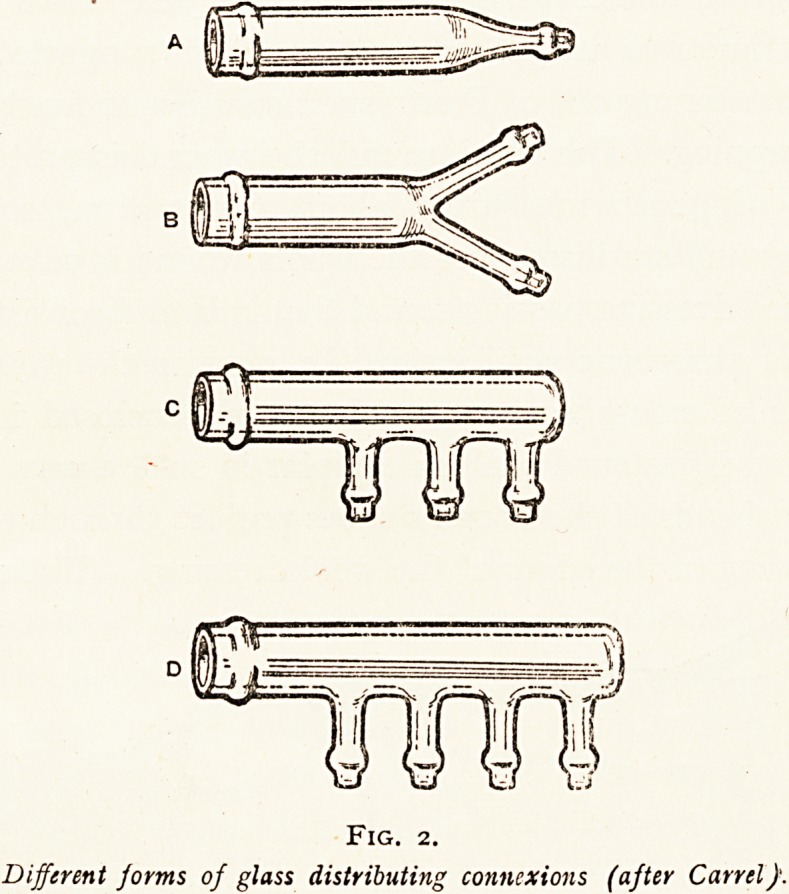


**Fig. 3. f3:**
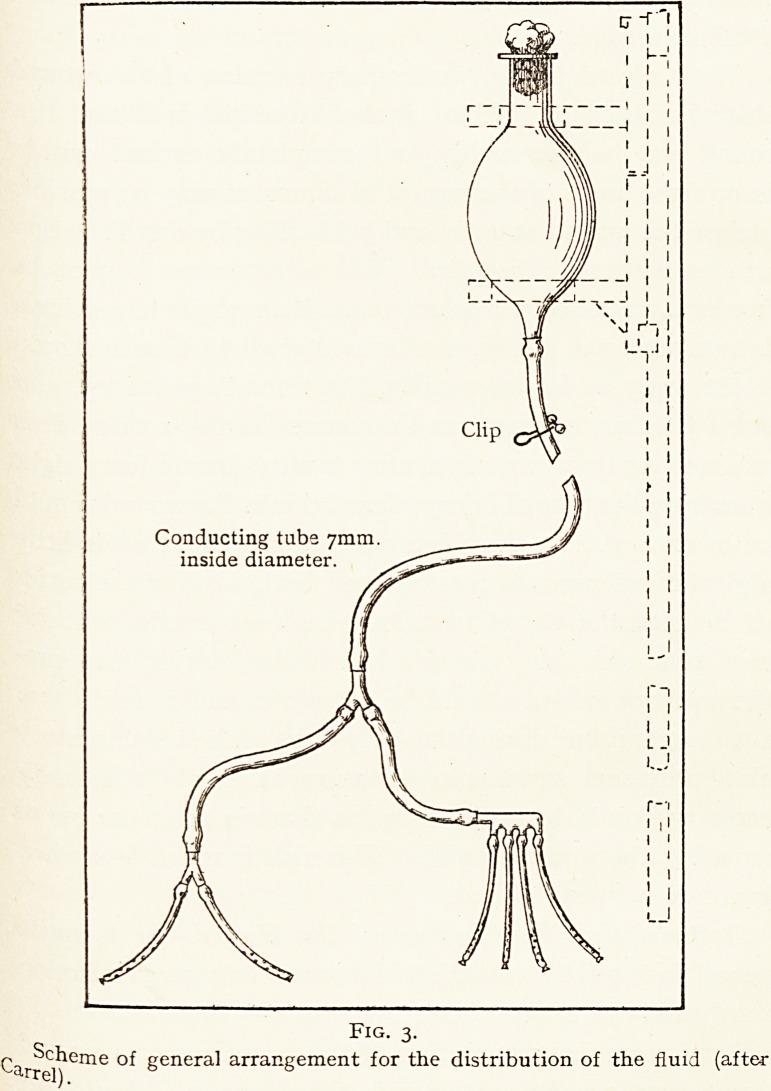


**Fig. 4. f4:**